# Value of scar imaging and inotropic reserve combination for the prediction of segmental and global left ventricular functional recovery after revascularisation

**DOI:** 10.1186/1532-429X-13-35

**Published:** 2011-07-25

**Authors:** Sigita Glaveckaite, Nomeda Valeviciene, Darius Palionis, Viktor Skorniakov, Jelena Celutkiene, Algirdas Tamosiunas, Giedrius Uzdavinys, Aleksandras Laucevicius

**Affiliations:** 1 Departament of Cardiovascular Medicine, Vilnius University; 2 Centre of Cardiology and Angiology, Vilnius University Hospitals Santariskiu klinikos, Santariskiu str. 2, 08661 Vilnius, Lithuania; 3 Clinic of Chest Diseases, Allergology and Radiology, Vilnius University, Lithuania; 4 Faculty of Mathematics and Informatics, Vilnius University, Lithuania

## Abstract

**Background:**

This study sought to prospectively and directly compare three cardiovascular magnetic resonance (CMR) viability parameters: inotropic reserve (IR) during low-dose dobutamine (LDD) administration, late gadolinium enhancement transmurality (LGE) and thickness of the non-contrast-enhanced myocardial rim surrounding the scar (RIM). These parameters were examined to evaluate their value as predictors of segmental left ventricular (LV) functional recovery in patients with LV systolic dysfunction undergoing surgical or percutaneous revascularisation. The second goal of the study was to determine the optimal LDD-CMR- and LGE-CMR-based predictor of significant (≥ 5%) LVEF improvement 6 months after revascularisation.

**Methods:**

In 46 patients with chronic coronary artery disease (CAD) (63 ± 10 years of age, LVEF 35 ± 8%), wall motion and the above mentioned CMR parameters were evaluated before revascularisation. Wall motion and LGE were repeatedly assessed 6 months after revascularisation. Logistic regression analysis models were created using 333 dysfunctional segments at rest.

**Results:**

An LGE threshold value of 50% (LGE50) and a RIM threshold value of 4 mm (RIM4) produced the best sensitivities and specificities for predicting segmental recovery. IR was superior to LGE50 for predicting segmental recovery. When the areas under the ROC curves is compared, the combined viability prediction model (LGE50 + IR) was significantly superior to IR alone in all analysed sets of segments, except the segments with an LGE from 26% to 75% (p = 0.08). The RIM4 model was not superior to the LGE50 model. A myocardial segment was considered viable if it had no LGE or had any LGE and produced IR during LDD stimulation. ROC analysis demonstrated that ≥ 50% of viable segments from all dysfunctional and revascularised segments in a patient predict significant improvement in LVEF with a 69% sensitivity and 70% specificity (AUC 0.7, p = 0.05). The cut-off of ≥ 3 viable segments was a less useful predictor of significant global LV recovery.

**Conclusions:**

LDD-CMR is superior to LGE-CMR as a predictor of segmental recovery. The advantage is greatest in the segments with an LGE from 26% to 75%. The RIM cut-off value of 4 mm had no superiority over the LGE cut-off value of 50% in predicting the segmental recovery. Patients with ≥ 50% of viable segments from all dysfunctional and revascularised had a tendency to improve LVEF by ≥ 5% after revascularisation.

## Background

Hibernating myocardium is normally defined as a viable and dysfunctional myocardium that improves in function following revascularisation [1]. The revascularisation of the hibernating myocardium results in an improvement of the regional and global left ventricular (LV) systolic function [2], reverse remodelling [3,4], increased survival [3] and a decrease in the composite end-point of myocardial infarction (MI), heart failure and unstable angina [5]. In contrast, patients with minimal or no evidence of myocardial viability appear to gain no benefit from revascularisation as compared to medical therapy [3].

Recently, the results of a viability substudy of the STICH (Surgical Treatment for Ischemic Heart Failure) trial were published [6]. This substudy was the first multicentre, non-blinded, randomised viability trial conducted to determine whether the presence of substantial myocardial viability influenced the likelihood of benefit from medical therapy plus coronary artery bypass graft surgery (CABG), as compared with medical therapy alone, in patients with CAD and LV dysfunction (LVEF ≤ 35%). This study, in contrast to a previous meta-analysis of the retrospective viability studies conducted by Allman et al. [3], did not find a significant interaction between myocardial viability status and medical treatment versus surgical treatment with respect to the rates of death from any cause or from cardiovascular causes or the rate of death or hospitalisation for cardiovascular causes. The conclusions regarding the optimal therapy for patients with CAD and LV dysfunction that can be drawn from the results of this viability substudy [6] are limited by a number of factors that are discussed in detail by Bonow et al. [6]. The viability analysis was based on identifying and quantifying the extent of viable myocardium in a binary fashion as (either having or not having substantial myocardial viability) by two different methods: single-photon-emission computed tomography (SPECT) and dobutamine echocardiography. The combination of the two different tests and the binary viability assessment fashion could be important limitations of this study. In addition, the findings of this substudy do not necessarily indicate that myocardial viability does not have a pathophysiological importance in patients with CAD and LV dysfunction. Instead, it is likely that some other variables in the analysis (e.g., LV volumes and LVEF) are causally determined by the extent of viable myocardium [6]. A cardiovascular magnetic resonance (CMR) viability assessment in this trial was not performed because of the limited data regarding outcomes in patients with chronic ischemic LV dysfunction studied by CMR.

Previous CMR studies have demonstrated that quantification of the transmural extent of LGE by CMR can be used to predict the likelihood of a recovery of myocardial function after revascularisation. However, in non-transmural scars (1% to 75%), only an intermediate likelihood of functional recovery (59.6% in LGE 1% to 25%, 41.8% in LGE 26% to 50% and 10.5% in LGE 51% to 75%) was found [7]. When the LDD stimulation was compared to scar imaging, LDD-CMR is superior to LGE-CMR in predicting the recovery of function after revascularisation [8]. This observation was most pronounced in segments with 1% to 74% transmural infarction [8]. It has been suggested that even though LGE-CMR depicts the area of myocardial fibrosis, it does not assess the functional state of the surrounding (potentially viable) myocardium, which can be normal, remodelled, hibernating, stunned or ischemic. Another small study (15 patients) conducted by Bove et al. [9] concluded that in segments with 1%-50% LGE transmurality, an improvement in wall thickening after revascularisation is better predicted by the response to LDD prior to revascularisation than by LGE. Kuehl et al. [10] suggested that the thickness of the non-contrast-enhanced and potentially viable myocardial rim surrounding the scar may be clinically useful for assessing myocardial viability. The functional state of unenhanced myocardial rim can be assessed using LDD-CMR, whereas the critical thickness of the scar surrounding unenhanced myocardial rim, which is needed to regain contractility after revascularisation, seems to be clinically useful in patients with an ischemic cardiomyopathy and regional wall thinning. This hypothesis was elegantly tested in 35 patients (LVEF 50 ± 11%) with chronic dysfunctional myocardium due to a chronic total occlusion by Kirschbaum et al. [11]. The authors of this study quantified IR using LDD-CMR in myocardial segments stratified according to the LGE transmurality, end-diastolic wall thickness and the thickness of the unenhanced rim and compared these with segmental wall thickening 6 months after a successful percutaneous coronary intervention (PCI). The results of this study indicated that in segments with an intermediate LGE (i.e., LGE transmurality between 25% and 75%), the measurement of baseline contractility of the unenhanced epicardial rim or simply baseline contractility of the wall (the authors assume that scar tissue does not contract) better identifies which segments maintain IR and recover after revascularisation than the LGE transmurality, end-diastolic wall thickness and the thickness of the unenhanced rim. The Kirschbaum study confirms that only the jeopardised dysfunctional myocardium of the unenhanced rim may have IR during LDD and recover after revascularisation; however, the normokinetic unenhanced rim has no IR and no recovery after successful PCI. The study authors [11] do not propose a clinical viability assessment algorithm. Thus, it remains unclear whether the additional use of LDD-CMR besides LGE-CMR is warranted and if it is enough to quantitatively determine only the rest function of the unenhanced rim in order to assess the presence of viable tissue. Furthermore, the value of the combination of different viability prediction parameters was not assessed in the aforementioned study.

All the aforementioned studies advance the concept that a more comprehensive approach to defining viability by CMR is warranted in clinical practice when the recovery of LV function is the desired endpoint. Thus, our primary goal was to prospectively and directly compare IR during LDD-CMR with the RIM and LGE as predictors of segmental functional recovery in patients with LV systolic dysfunction undergoing surgical or percutaneous revascularisation. In the current study, we prospectively tested the hypothesis that the addition of LDD-CMR and quantification of IR or additional measurement of the RIM in segments with 1 to 75% LGE would improve the predictive value for the recovery of LV segmental function after revascularisation in patients with ischemic LV dysfunction. To the best of our knowledge, this comparison has not been studied in patients undergoing either surgical or percutaneous revascularisation. Thus, the comparisons have not been made in a patient cohort that most accurately represents real clinical practice. The second goal of this study was to determine the optimal LDD-CMR- and LGE-CMR-based predictor of significant (≥ 5%) LVEF improvement 6 months after revascularisation. Surprisingly, the combined LDD-CMR- and LGE-CMR-based predictor of significant improvement of global LVEF has not been investigated thus far.

## Methods

### Patients and Study Design

A prospective evaluation of the different CMR parameters for predicting LV segmental and global functional recovery was performed in 46 patients (63 ± 10 years old, 3 with previous CABG, 35 with three-vessel disease, 3 with one-vessel disease) with LV systolic dysfunction (LVEF 35 ± 8%) before they underwent surgical (n = 34) or percutaneous (n = 12) revascularisation. Sixty patients without contraindications for CMR were screened for the following inclusion criteria: (1) CAD (> 70% stenosis in one or more major epicardial vessels), scheduled for a revascularisation procedure; (2) LVEF ≤ 45%; (3) at least two adjacent segments with wall motion abnormalities at rest; and (4) no infarction or revascularisation within the last two months. Patients were included in the study only after a successful and complete coronary revascularisation. Of the 14 patients who did not complete the study, 3 decided not to undergo the repeated CMR scan or were lost during follow-up; 7 had significant periprocedural injury (new LGE zones on repeated CMR scans and clinically proven periprocedural myocardial infarction (MI) or MI between both scans); 3 had pacemakers or defibrillators implanted in the period between the MR scans; and 1 was excluded because of dilated cardiomyopathy with secondary CAD. None of the patients were excluded from the study for technical reasons or image quality.

The mean interval between CMR and revascularisation was 12 ± 13 days, and none of the patients presented clinical evidence of infarction during this period. The mean interval between MI and the first CMR was 3.6 years. In 46 patients, the extent of regional contractility and LGE were determined repeatedly by CMR 28 ± 4 weeks (6 months) after revascularisation.

The study was approved by the Lithuanian Bioethics Committee (Nr. 17), and informed written consent was obtained from each patient prior to inclusion in the study.

### CMR protocol

All the CMR examinations were performed using a 1.5 Tesla MR scanner (Avanto, Siemens Medical Solutions, Erlangen, Germany) using prospective gating. Steady-state free precession cine CMR was performed with breath holding. Four-, 3- and 2-chamber views, as well as a short axis stack covering the left ventricle every 8 mm without a gap, were acquired at rest and at the after each dose of dobutamine (5 and 10 μg/kg/min) (TE/TR/flip angle 1.22 ms/63 ms/65 degrees, FOV 250 mm, voxel size 1.9 × 1.3 × 8 mm, matrix size 109 × 192). After revascularisation, only rest images were acquired using the same technique.

Ten to fifteen minutes after infusing 0.15 mmol/kg of the commercially available gadolinium-based contrast agent (gadopentetate dimeglumine or gadodiamide), an inversion recovery gradient-echo sequence (TE/TR/flip angle 3.2 ms/700 ms/25 degrees, FOV 400 mm, matrix size 156 × 256) was performed in the same planes as the cine images with an inversion time (240 to 330 ms) chosen to reduce the signal from normal myocardium. The typical voxel size was 2.1 × 1.6 × 8 mm. Angulation was kept constant for a short-axis and LGE imaging to enable a match between the LGE and wall motion images.

### Post-processing Analysis

We analysed the cine images and contrast-enhanced images using a model in which the LV was divided into 17 segments [12]. The wall motion was graded as 1 (normal), 2 (mild hypokinesia), 3 (severe hypokinesia), 4 (akinesia) or 5 (dyskinesia) by 2 blinded investigators. Discordant assessments were jointly reviewed. For the patients undergoing percutaneous revascularisation, segments were considered to be undergoing revascularisation according to the scheme suggested by Haug [13]. The LV apical segment was assigned to a specific coronary artery territory according to the vessel anatomy on a conventional angiogram. For the global LV functional analysis, all short-axis slices from the base to the apex at rest and during administration of dobutamine (10 μg/kg/min) were analysed with Argus software (Siemens) by two independent experienced observers. The wall motion score index (WMSI) was calculated by dividing the sum of scores by the number of segments per patient. The LV sphericity index (SI) was measured by dividing the length of the LV from the apex to the mitral annulus by the width of the LV at the basal aspect of the papillary muscles in the end-diastolic apical four-chamber view. An absolute change in LVEF ≥ 5% 6 months after revascularisation was considered to be significant. When predicting significant LVEF improvement, a segment was considered viable if it had no LGE or had any LGE and produced IR during LDD stimulation. The number of viable segments divided by the total number of dysfunctional and revascularised segments in the patient was expressed as a percentage that was used together with the absolute number of viable segments to predict significant LVEF improvement. We compared two groups: responders (i.e., patients with significant LVEF improvement 6 months after revascularisation) and non-responders (i.e., patients without significant LVEF improvement (improvement of LVEF < 5%)).

The extent of LGE within each segment and the RIM were also measured by the two independent experienced investigators on short-axis, contrast enhanced CMR images. Contrast enhanced pixels were defined as those with image intensities > 2 SD above the mean of image intensities in a remote myocardial region in the same image. LGE was assessed on a 5-grade scale [7] and analysed quantitatively by dividing the hyperenhanced area, as measured by computer-assisted tracings, by the total area in each segment before being expressed as a percentage. The thickness of the unenhanced rim was defined as the mean wall thickness of the nonenhanced area of a segment. Only the dysfunctional segments detected on the first MR scan and those without an increase in the LGE area on the second MR scan were analysed. To calculate the mass of the LGE, we assumed a specific gravity of 1.05 g/cm^3^. Interobserver variability for the transmural grading was checked for 10 patients by 2 blinded experienced observers. The observers had a good level of agreement (kappa = 0.88). The total LGE score per patient was calculated by dividing sum of the LGE scores by the number of segments.

An improvement in wall motion at follow-up by at least 1 grade with the exception of improvement from grade 5 to grade 4 was regarded as functional recovery or viability of the segment. The LDD-CMR was regarded as indicative of viability or IR when there was an improvement of 1 wall motion grade at either the 5 or 10 μg/kg/min dose. All reviewers of the segmental wall motion, LDD-CMR, LGE and functional recovery were blinded to each other and to the clinical data of the patients. All discordant assessments were jointly reviewed.

### Statistical analysis

To compare the values of different CMR parameters for predicting segmental functional recovery, the usual characteristics, such as sensitivity, specificity, positive predictive value (PPV) and negative predictive value (NPV) were calculated. Using a logistic regression model, we identified the threshold values that produced the optimal sensitivities and specificities. Being optimal did not mean that this threshold produced the highest accuracy. The difference between sensitivity and specificity was also considered (e.g., a threshold producing 99% sensitivity and 10% specificity was not treated as the best, even if its accuracy was the highest). Furthermore, we built several logistic regression models to predict myocardial viability using IR, LGE50 and RIM4 values. For LGE50 and RIM4, the calculations were performed using binary variables, which were assigned a value of 1 if the measurement exceeded a threshold value and a value of 0 otherwise (e.g., LGE50 = 1 when LGE > 50 and LGE50 = 0 when LGE ≤ 50). As we wanted not only to test whether single parameters perform differently but also to find out whether there is rationale for using a combination of several methods, 5 different logistic regression models were created. In all of the viability models, functional improvement after revascularisation acted as a dependent variable. Meanwhile, the other above-mentioned CMR parameters acted as independent variables. All independent variables were statistically significant. To find out which method had the best predictive ability, we measured the areas under the receiver operating curves (ROC) of the five different logistic regression models.

The different baseline and follow-up characteristics of patients with and without significant improvement in LVEF 6 months after revascularisation were compared. The values from both patient groups were expressed as mean ± SD. The effect of revascularisation was compared using a Wilcoxon signed-rank test. The continuous variables that were not distributed normally were compared by using a nonparametric test. The variables that differed significantly between groups were included in a forward stepwise (Wald) logistic regression analysis to determine the best independent predictor of significant LVEF improvement. The ROC analysis was performed to validate the variables with the best predictive ability. The predictor of global functional recovery was treated superior to the other methods if its area under the ROC curve (AUC) was significantly greater.

All calculations were performed using SPSS 16.0 and StAR [14] software. A p-value < 0.05 was considered statistically significant.

## Results

Forty-six patients underwent a successful and complete revascularisation procedure. A significant improvement in LVEF ≥ 5% was demonstrated in 36/46 (78%) patients, and the baseline characteristics of patients with and without significant improvement in LVEF are given in Table 1. Only the functional LV parameters before revascularisation differed significantly between the groups. Patients in the nonresponder group had a significantly lower LVEF, greater LV volume indexes and greater wall motion score indexes.

**Table 1:** The baseline characteristics of patients with and without significant improvement in LVEF

Baseline characteristics	All patients n = 46	Responders n = 36	Non-responders n = 10	p value
**Age (yrs)**	63 ± 10	64 ± 10	60 ± 8	0.183
**Female**	5	5	0	0.570
**GFR ml/min**	91 ± 33	88 ± 34	101 ± 27	0.184
**BSA (m^2^)**	2 ± 0.2	2 ± 0.3	2 ± 0.1	0.516
**Hypertension**	42	32	10	0.562
**Diabetes mellitus**	7	5	2	0.786
**Previous documented MI**	42	32	10	0.562
**NYHA functional class**	2.7 ± 0.8	2.6 ± 0.8	2.7 ± 1.0	0.634
**CABG**	34	26	8	0.512
***ONBEAT***	7	6	1	0.518
***ONSTOP***	27	20	7	0.518
***Nr. of distal anastomoses***	2.3 ± 1.6	2.2 ± 1.6	2.6 ± 1.6	0.422
***LIMA***	27	22	5	0.195
**PCI**	12	10	2	-
**Beta-blocker**	40	31	9	1.000
**ACE inhibitor**	33	27	6	0.351
**Statin**	37	28	9	0.389
**Duration between revascularisation and follow-up CMR (weeks)**	28 ± 4	28 ± 3	29 ± 8	0.704
**LVEF (%)**	35 ± 8	36 ± 8	32 ± 7	0.041
**LVEF 30% or less**	10	7	3	0.474
**LV EDVI (ml/m^2^)**	95 ± 35	90 ± 35	114 ± 30	0.035
**LV ESVI (ml/m^2^)**	62 ± 28	58 ± 28	77 ± 22	0.009
**LV SI**	0.56 ± 0.1	0.55 ± 0.1	0.6 ± 0.1	0.068
**WMSI**	1.9 ± 0.4	1.8 ± 0.4	2.2 ± 0.4	0.003
**Total LGE score**	1.0 ± 0.6	0.9 ± 0.6	1.2 ± 0.7	0.170
**LGE mass (g)**	31 ± 21	29 ± 21	36 ± 21	0.311

A responder was defined as a patient with an improvement in LVEF ≥ 5% after revascularisation. GFR, glomerular filtration rate; BSA, body surface area; MI, myocardial infarction; CABG, coronary artery bypass graft surgery; ONBEAT, on-pump beating heart CABG; ONSTOP, conventional cardioplegic arrest CABG; LIMA, left internal mammary artery; PCI, percutaneous coronary intervention; CMR, cardiovascular magnetic resonance; LVEF, left ventricular ejection fraction; EDVI, end-diastolic volume index; ESVI, end-systolic volume index; LGE, late gadolinium enhancement; WMSI, wall motion score index; SI, sphericity index.

**Figure 1:**
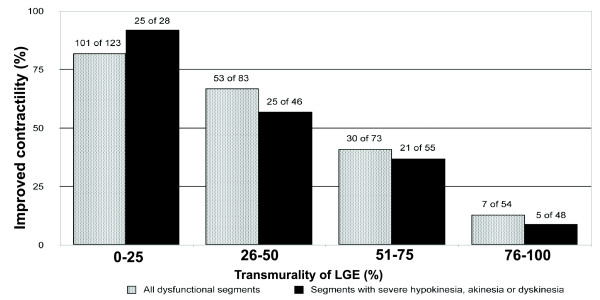
**Relationship between LGE before revascularisation and the likelihood of improved segmental contractility after revascularisation**. Data are shown for all 333 dysfunctional segments and separately for the 177 segments with at least severe hypokinesia before revascularisation. For both analyses, there was an inverse relationship between the LGE and the likelihood of improvement in contractility.

Overall, 333 (43%) of the 782 myocardial segments analysed had abnormal contractility and underwent successful revascularisation. A functional recovery was observed in 191 (57%) segments, but the remaining 142 segments (43%) showed no signs of functional recovery after revascularisation. The functional recovery of the myocardium decreased with increasing LGE transmurality (82% segments with functional recovery in LGE 0% to 25%, 64% in LGE 26% to 50%, 41% in LGE 51% to 75% and 13% in LGE > 76% were found) (Figure 1). A similar trend in segmental functional recovery was observed in 177 segments with severe hypokinesia, akinesia or dyskinesia (89% segments with functional recovery in LGE 0% to 25%, 54% in LGE 26% to 50%, 38% in LGE 51% to 75% and 10% in LGE > 76%) (Figure 1). Sixty-two per cent of segments with functional recovery were observed in the group with an end-diastolic wall diameter > 5.5 cm; however, only 41% of segments with an end-diastolic wall diameter ≤ 5.5 cm recovered after revascularisation.

### Prediction of myocardial regional functional recovery after revascularisation using different CMR parameters

Based on segmental functional recovery, 6 months after revascularisation, we calculated the prognostic value of different CMR parameters and threshold values for the LGE and the RIM that could be used in practice to distinguish between viable and nonviable segments (Table 2). We can clearly see that the sensitivities of the LGE and the IR are comparable, but the specificity of the IR is higher than the specificity of the LGE. The RIM specificity and sensitivity is slightly lower than the respective values for the IR.

**Table 2:** The prognostic value of the three different CMR viability parameters

Parameter	Sensitivity (%)	Specificity (%)	PPV (%)	NPV (%)	Threshold value	No. of analysed segments
**LGE**	80	62	73	71	50%	333
**RIM**	77	72	69	80	4 mm	214
**IR**	80	78	83	75	-	333

The LGE and the RIM threshold values were calculated using the logistic regression model. PPV, positive predictive value; NPV, negative predictive value; no., number.

**Figure 2:**
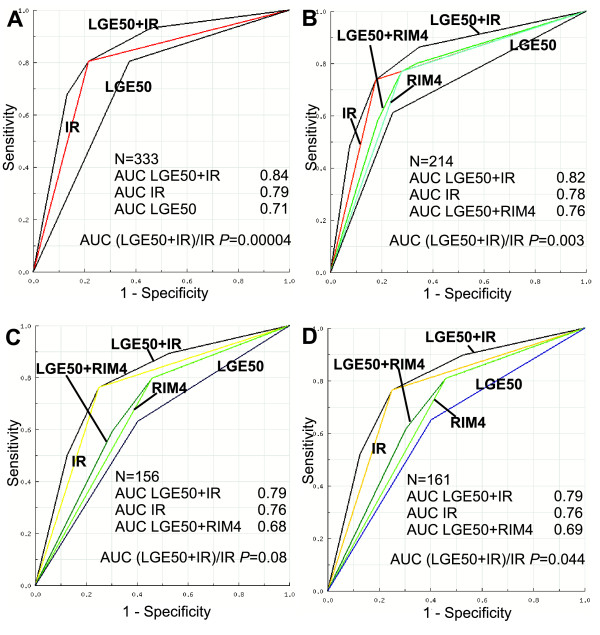
**ROC curves: the logistic regression model combining LGE50 and IR compared to IR alone and LGE50 alone**. The AUC value for LGE50 + IR was significantly higher than IR alone or LGE50 alone in all analysed segments (A); the AUC value for LGE50 + IR was significantly higher than IR alone or LGE50 alone in the segments containing any degree of LGE (B); the AUC value for LGE50 + IR differs insignificantly from the IR alone in the segments with 26% to 75% LGE (C); and the difference between LGE50 + IR and IR alone is significant in segments with 1% to 75% LGE (D).

To prospectively and directly compare the predictive value of the IR, RIM and LGE, we used binary variables for the LGE and the RIM (LGE50, 50% cut-off and RIM4, 4 mm cut-off). For a more detailed description of notations, see the statistical analysis section. We compared the areas under the ROC curves obtained using five different logistic regression models (Figure 2). When the areas under the ROC curves were compared, the combined viability prediction model (LGE50 + IR) was superior to IR alone in all analysed sets of segments except for segments with an LGE from 26% to 75% (p = 0.08). The IR alone was statistically significantly superior to the LGE50 alone in all the analysed sets of segments (p = 0.0066 in all analysed segments and p = 0.043 in segments with LGE from 26% to 75%).

Taking into account only the segments with any degree of LGE (Figure 2B, C, D), the areas under the ROC curves for IR alone, RIM4 alone and LGE50 + RIM4 differed insignificantly. The AUC of the combined (LGE50 + RIM4) model differed significantly from the LGE50 alone model in all subsets of segments with any degree of LGE; however, the areas under the ROC curves of RIM4 alone and LGE50 alone were comparable in the segments with 26% to 75% and 1% to 75% LGE (p = 0.15 and p = 0.18, respectively). A statistically significant difference in the AUC between the two combined models (LGE50 + IR and LGE50 + RIM4) was observed, with the LGE50 + IR viability prediction model being superior. There was no significant difference between IR alone and LGE alone in the segments with LGE ≥ 76%.

When comparing the above-mentioned viability prediction parameters in patients with LVEF ≤ 30% and > 30%, IR was observed to be superior to LGE50 in the group with LVEF > 30% (p < 0.00001) but not in the group with LVEF ≤ 30% (p = 0.29). The same significant superiority of the combined viability prediction model (LGE50 + IR) over the IR only model was noticed in both patient groups. In patients with LVEF ≤ 30%, using the combined viability prediction model (LGE50 + IR), the percentage of correct predictions for hibernating myocardium was 79%, compared to 81% for patients with LVEF > 30%.

### Prediction of global left ventricular functional recovery after revascularisation

Overall, the mean improvement in global ventricular function 6 months after revascularisation was 11 ± 7%. In the group with significant LVEF improvement, the mean NYHA functional class was improved by 1 class, whereas in the group without significant LVEF improvement, the mean NYHA functional class remained unchanged. At follow-up, none of the patients had angina pectoris of more than class I CCS.

**Figure 3:**
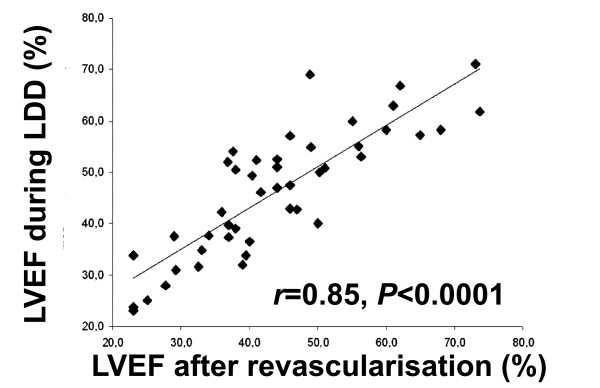
**Correlation between LVEF measured during LDD administration and LVEF 6 months after revascularisation**.

There was a strong inverse correlation between the baseline WMSI and LVEF 6 months after revascularisation (r = -0.75, p < 0.0001); however, the correlation between the mass of LGE to LVEF change and the correlation between the mass of LGE and the LVEF after revascularisation was weak (r = -0.35 and r = -0.39, respectively). Interestingly, we found an excellent correlation between LVEF measured during administration of dobutamine (10 μg/kg/min) and LVEF 6 months after revascularisation (Figure 3).

Six months after revascularisation, we observed a significant difference between the responder and nonresponder groups in LVEF (50 ± 11% vs. 33 ± 6%, respectively, p < 0.001) and LV sphericity index (0.5 ± 0.1 vs. 0.6 ± 0.1, respectively, p = 0.048) and a borderline significant difference in the number of segments with functional recovery (4.4 ± 2.6 vs. 2.9 ± 2.1, respectively, p = 0.077). Taking into account the changes of LV functional parameters within each group, both groups demonstrated significant improvement in WMSI; however, a significant improvement of ESVI at follow-up was observed only in the responder group (Table 3). Changes in the other LV parameters (i.e., mitral valve regurgitation fraction, EDVI and sphericity index) after revascularisation were insignificant in both groups.

**Table 3:** The dynamic changes in LV function after revascularisation within groups of patients with and without significant improvement in LVEF

Responders	Baseline	Follow-up	Mean diff. ± SD	p value
**EDVI**	90 ± 35	88 ± 24	2.1 ± 26	0.326
**ESVI**	58 ± 28	46 ± 21	12 ± 13	<0.001
**EF (%)**	36 ± 8	50 ± 11	14 ± 6	<0.001
**WMSI**	1.8 ± 0.4	1.5 ± 0.4	0.3 ± 0.3	<0.001

**Non-responders**				

**EDVI**	114 ± 30	111 ± 26	3.0 ± 29	0.492
**ESVI**	77 ± 22	74 ± 19	3.0 ± 20	0.557
**EF (%)**	32 ± 7	33 ± 6	1.4 ± 2.4	0.105
**WMSI**	2.2 ± 0.4	2.0 ± 0.4	0.2 ± 0.2	0.020

Same abbreviations as used in Table 1.

To assess the best CMR-based predictors of significant LVEF improvement, we used variables that differed significantly between the responders and nonresponders at baseline (Table 1). Using forward stepwise logistic analysis, we found that EDVI (p = 0.79), ESVI (p = 0.76) and WMSI (p = 0.26) are not good predictors of significant LVEF improvement.

**Figure 4:**
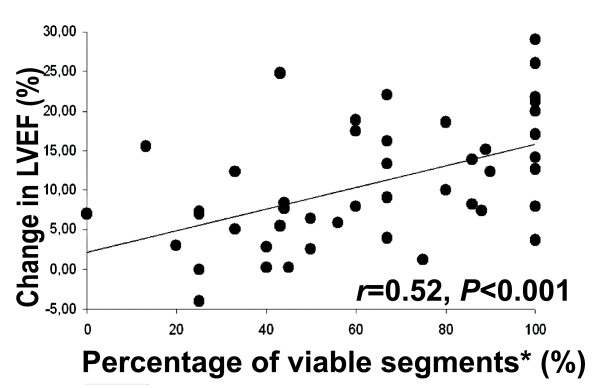
**Correlation between the percentage of viable segments and the change in LVEF 6 months after revascularisation**. *Percentage of viable segments is defined as the number of viable segments in a patient divided by all dysfunctional and revascularised segments and is expressed as a percentage.

**Figure 5:**
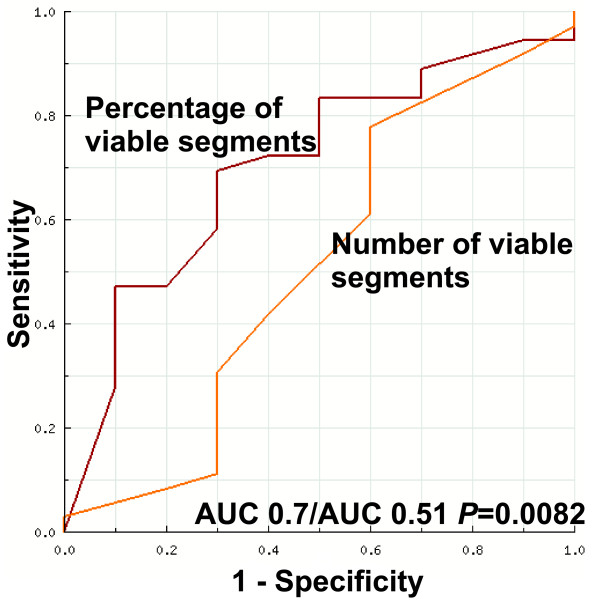
**The areas under the ROC curves for the percentage of viable segments and the number of viable segments for predicting significant improvement in global LV function after revascularisation**. Definitions of the terms are in the text.

Additionally, we compared two other parameters: the absolute number of viable segments in a patient and the percentage of viable segments from all dysfunctional and revascularised segments in a patient. A viable segment was defined as a segment without any LGE or with any LGE and having IR during LDD-CMR. The relationship between the percentage of viable segments and the change in LVEF was relatively close to linear (Figure 4). Using ROC analysis, the AUC for the percentage of viable segments was 0.7 (p = 0.05) compared to AUC 0.52 for the number of viable segments (p = 0.94) (Figure 5). This finding shows that the absolute number of viable segments is inferior to the percentage of viable segments for predicting significant LVEF improvement. An additional ROC analysis was used to define a threshold for the percentage of viable segments in a patient that had the optimal sensitivity and specificity for predicting global function recovery. The application of a cut-off value of ≥ 50% viable segments yielded a 69% sensitivity and a 70% specificity (AUC 0.7, p = 0.054). A cut-off of 3 viable segments produced lower diagnostic value, with a sensitivity of 78% and a specificity of 40%.

## Discussion

### Prediction of regional functional recovery

The decreasing likelihood of functional recovery with more extensive scarring found in the present study confirms the prognostic importance of scarred myocardium, which is consistent with previous studies [7-9]. The high percentage (82%) of segments with no or minimal scarring (LGE ≤ 25%) in our cohort recovered 6 months after revascularisation, which agrees with results of previous studies conducted by Selvanayagam et al. [15] and Bondarenko et al. [16]. The high percentage of recovered segments in our cohort confirms that a 6-months post-revascularisation period was sufficient for almost complete recovery.

Using an LGE threshold value of 50%, we can roughly differentiate patients in whom recovery of regional myocardial function is likely or unlikely, with a sensitivity of 80% and a specificity of 62%. An RIM value of 4 mm can predict the recovery of hibernating myocardium with a sensitivity and specificity of approximately 77% and 72%, respectively. Although the sensitivities of LGE 50 and IR are comparable, the IR specificity is higher (78%), which is consistent with previous studies [8].

Additionally, comparing the areas under the ROC curves, we found that IR alone is statistically significantly superior to LGE50 alone in predicting segmental functional recovery in all analysed sets of segments. The latter finding does not contradict the findings of a previous study conducted by Wellnhofer et al. [8]. Interestingly, the combined viability prediction model (LGE50 + IR) was significantly better than IR alone in all analysed subsets of segments, except in segments with LGE from 26% to 75%. These results confirm the large advantage of LDD-CMR versus LGE-CMR in segments with 26% to 75% LGE. In patients with an intermediate LGE from 1% to 75% and from 26% to 75%, the addition of the IR improved the correct predictions of the hibernating myocardium from 65% to 76% and from 63% to 76%, respectively.

In ischemic cardiomyopathy with regional wall thinning, the addition of RIM measures to LGE can be useful, but according to our findings, the RIM did not give much more information than LGE, especially in segments with LGE from 1% to 75% or from 26% to 75%. As we expected, the correlation coefficient between the RIM and LGE is high (r = -0.81), and the AUC values are comparable because these two parameters carry virtually the same information regarding scar transmurality. In contrary, a study conducted by Ichikawa et al. [17] confirmed that the thickness measurement of nonenhanced myocardium, compared with measurement of LGE per cent, possessed better diagnostic accuracy for predicting improved systolic wall thickening from acute to chronic MI state in dysfunctional segments. The latter findings cannot be directly compared to our findings because [17] their study focused on a stunned and timely reperfused myocardium in patients with relatively preserved LV function, whereas our study focused on hibernated and revascularised myocardium in patients with moderately to severe LV function impairment. Additionally, a study conducted by Kirschbaum et al. [11] confirmed that the baseline function of the unenhanced myocardial rim is more important than the rim thickness in predicting the presence of inotropic reserve in segments with intermediate LGE. Our findings confirm the fact that in segments with intermediate LGE, functional assessment of the RIM (i.e., assessment of the inotropic reserve during LDD) is more important than measuring RIM thickness. Thus, it is possible to assess the inotropic reserve during LDD or to measure the baseline systolic wall thickening of the RIM (as performed in [11]) in segments with intermediate LGE, but further studies directly comparing these two approaches as predictors of functional recovery after revascularisations are needed.

Interestingly, the IR alone model and the combined viability prediction model (LGE50 + RIM4) differed insignificantly in segments with any degree of contrast enhancement, especially in segment subsets with LGE 1-75% and 26-75%; however, the LGE50 + IR model was significantly superior to the LGE50 + RIM4 model. This suggests that the additional value of IR assessment is greater than that of RIM4 in segments with intermediate LGE.

The results of the aforementioned analysis indicate that the addition of LDD-CMR to LGE-CMR improves viability prediction when all dysfunctional segments, including and excluding those without any contrast enhancement, are analysed. IR is superior to LGE50 in predicting hibernating myocardium in all sets of segments. Taking into account the fact that most of the segments (approximately 82%) with LGE ≤ 25% recover and that most of the segments (approximately 87%) with LGE ≥ 76% do not recover function after revascularisation, the evaluation of additional viability parameters besides LGE seems to have little additional value in this subset of segments. Thus, the addition of LDD-CMR seems to have the biggest additional value in segments with 26% to 75% LGE, whereas measuring of the RIM thickness has no superiority over LGE50 in this LGE subset.

The high percentage (79%) of correct predictions for hibernating myocardium in the patient group with EF ≤ 30% confirms the hypothesis that CMR is very suitable for viability prediction, especially in patients with severe LV dysfunction, in whom echocardiographic methods of viability prediction are less accurate [18].

Taking into account the higher predictive value of IR compared with LGE50, it is possible to use LDD-CMR instead of LGE-CMR to assess viability in selected patients with severely reduced renal function (GFR <30 ml/min) to avoid the risk of nephrogenic systemic fibrosis.

### Prediction of global functional recovery

We found an excellent correlation between LVEF during LDD and LVEF 6 months after revascularisation (r = 0.85, p < 0.0001). This suggests that by measuring LVEF by CMR during LDD administration, it is possible to predict the absolute LVEF 6 months after successful revascularisation.

At baseline, we observed significant differences between the groups with and without significant LVEF improvement regarding LV volume indexes, WMSI and LVEF. Patients in the nonresponder group had more remodelled left ventricles at baseline, lower LVEF, higher LV volume indexes and higher WMSI. These baseline factors could contribute to the fact that the 6-month follow-up period could be too short for significant inverse remodelling in such ventricles. We observed insignificant changes in the LV volume index, sphericity index and WMSI at follow-up. However, patients in the responder group showed a trend to inverse remodelling of the LV. They experienced a significant decrease in ESVI and WMSI and a marked increase in LVEF.

Previous studies have demonstrated that a substantial amount of the jeopardised myocardium needs to be present to result in an improvement of LVEF after revascularisation. In previous studies, the setting of a cut-off level of ≥ 4 dysfunctional and viable segments (representing approximately 25% of the left ventricle) assessed by echocardiography yielded the highest diagnostic accuracy to predict improvement in LVEF [19,20]. The present study demonstrates that the absolute number of viable segments has a lower predictive value for global LV functional recovery than the percentage of viable segments. The weak predictive value of the number of viable segments was recently reported by other investigators (i.e., Pegg et al.) [21]. As we were basing our experiments on a different study design and relying on our segmental functional recovery prediction results, we incorporated LGE-CMR and LDD-CMR data. Our results concerning global functional recovery prediction indicate that the best predictor of significant LVEF improvement 6 months after revascularisation in our cohort was the percentage of viable segments from all dysfunctional and revascularised segments in a patient. The cut-off value ≥ 50% predicts significant LVEF improvement with 69% sensitivity and 70% specificity (AUC 0.70, p = 0.054). Although the p-value is of borderline significance, we think that the value could be influenced by the small sample size, especially in the nonresponder group. The predictor of global functional recovery in our study had a lower predictive value than the predictor used in a study conducted by Pegg et al. [20]; this could be explained by a different definition of significant LVEF improvement (i.e., ≥ 3% change in EF [20] versus ≥ 5% change in our cohort). Further studies are warranted to confirm our findings. However, these findings could be relevant for clinicians making decisions regarding revascularisation in patients with impaired LV function in everyday practice. Our findings and the results of the study conducted by Pegg et al. [21] raise questions regarding the definition of substantial myocardial viability in further clinical viability studies. The above-mentioned STICH viability substudy failed to demonstrate a significant interaction between myocardial viability status and medical versus surgical treatment with respect to the mortality or the rate of death or hospitalisation for cardiovascular causes. The primary reason for this failure was that the absolute number of viable segments was used in the STICH trial instead of other, more sophisticated definitions of substantial amount of viable myocardium (e.g., percentage of viable segments, as in present study).

### Clinical implications

For clinical use, we propose to initially perform LGE-CMR and add the LDD-CMR just after the LGE-CMR only in patients with LGE from 1% to 75%, as the addition of LDD significantly improves viability prediction in this subset of patients. The measurement of RIM thickness in the segments with any degree of LGE does not give more information than LGE. Revascularisation, in cases when patients have no angina pectoris and the target is the improvement of heart failure symptoms, should be performed when there is a substantial amount of viable myocardium, (50% or more viable segments from all dysfunctional and revascularised segments).

### Limitations

The major limitation of present study is the small sample size. However, this sample size is comparable to previously published studies that used LGE-CMR and LDD-CMR in patients with chronic ischemic heart disease undergoing revascularisation [8,9,11]. In our study, the verification of functional recovery was performed at 6 months after revascularisation, and this time period seemed sufficiently late in view of the high percentages of correct predictions. However, the use of a single evaluation for ventricular function in the short period after revascularisation may lead to an underestimation of the true rate of functional recovery because the time course of full recovery may be up to 24 ± 12 months [16]. However, with a longer follow-up period, LV function could be strongly influenced by late graft failure or stent restenosis [16]. Even if technically successful, coronary revascularisation may be incomplete, particularly in patients with extensive atherosclerosis and diffuse disease. Although restenosis/graft occlusion was excluded through invasive procedures in nine patients (20%), their non-invasive follow-up revealed that they were free of symptoms or signs indicating recurrent ischemia or major adverse cardiac events. Not one patient from our study group manifested any new wall motion abnormalities at follow-up. Furthermore, myocardial segments with new LGE zones, which were observed in 7 patients, were excluded from the analysis. The visual assessment of wall motion is also a limitation of the present study. A quantitative assessment of intramyocardial deformation or strain during LDD-CMR with rapid post-processing algorithms is a promising technique for the more accurate prediction of functional recovery, but future studies are needed before inclusion of these techniques into routine clinical practice.

## Conclusions

LGE-CMR and LDD-CMR provide complementary information regarding myocardial viability, and a combination of both techniques is valuable for a more accurate prediction of viability. LDD-CMR is superior to LGE-CMR as a predictor of segmental functional recovery and does not depend on the transmurality of the scar. In segments with an LGE from 26% to 75%, LDD-CMR is not inferior to the combination of LDD-CMR and LGE-CMR; thus, the greatest advantage of IR is in segments with LGE from 26% to 75%. The RIM did not give much more information than LGE. When defining viability as the absence of LGE or the presence of IR in the case of any degree of LGE, patients with ≥ 50% of viable segments from all dysfunctional and revascularised segments have a tendency to improve LVEF ≥ 5% after revascularisation. There are trends towards LV reverse remodelling in the group with significant LVEF improvement after revascularisation. By measuring LVEF during LDD administration, it is possible to predict the absolute value of LVEF 6 months after revascularisation.

## List of abbreviations

ACE: angiotensin-converting enzyme; AUC: area under the curve; BSA: body surface area; CABG: coronary artery bypass graft surgery; CCS: Canadian Cardiovascular Society; CMR: cardiovascular magnetic resonance; EF: ejection fraction; GFR: glomerular filtration rate; IR: inotropic reserve; LAD: left anterior descending artery; LDD: low-dose dobutamine; LIMA: left internal mammary artery; LGE: late gadolinium enhancement; LV: left ventricle; MI: myocardial infarction; NPV: negative predictive value; NYHA: New York Heart Association; ONBEAT: on-pump beating heart coronary artery bypass graft surgery; ONSTOP: conventional cardioplegic arrest coronary artery bypass graft surgery; PCI: percutaneous coronary intervention; PPV: positive predictive value; RIM: thickness of the non-contrast-enhanced myocardial rim surrounding the scar; ROC: receiver operating curve; SPECT: single-photon-emission computed tomography; SSFP: steady-state free precession sequence; WMSI: wall motion score index

## Competing interests

The authors declare that they have no competing interests.

## Authors' contributions

SG made substantial contributions to the study conception, design, data analysis and interpretation, wrote the manuscript draft and revised it critically according the suggestions of the other authors and the JCMR reviewers; NV made substantial contributions to the study design, was involved in data acquisition and critically revised the manuscript draft for important intellectual content; DP was involved in data acquisition and critically revised the manuscript draft for important intellectual content; JC critically revised the manuscript draft for important intellectual content; VS performed the statistical data analysis and interpretation and critically revised the manuscript draft for important intellectual content; AL made substantial contributions to the study conception and design, critically revised the manuscript draft for important intellectual content and gave final approval of the version to be published; GU critically revised the manuscript draft for important intellectual content. All the authors read and approved the final manuscript.
